# Swab2know: An HIV-Testing Strategy Using Oral Fluid Samples and Online Communication of Test Results for Men Who Have Sex With Men in Belgium

**DOI:** 10.2196/jmir.4384

**Published:** 2015-09-01

**Authors:** Tom Platteau, Katrien Fransen, Ludwig Apers, Chris Kenyon, Laura Albers, Tine Vermoesen, Jasna Loos, Eric Florence

**Affiliations:** ^1^ Institute of Tropical Medicine Department of Clinical Sciences Antwerp Belgium; ^2^ Institute of Tropical Medicine Department of Public Health Antwerp Belgium

**Keywords:** HIV, men who have sex with men, MSM, self-sampling, oral fluid, online testing

## Abstract

**Background:**

As HIV remains a public health concern, increased testing among those at risk for HIV acquisition is important. Men who have sex with men (MSM) are the most important group for targeted HIV testing in Europe. Several new strategies have been developed and implemented to increase HIV-testing uptake in this group, among them the Swab2know project.

**Objective:**

In this project, we aim to assess the acceptability and feasibility of outreach and online HIV testing using oral fluid samples as well as Web-based delivery of test results.

**Methods:**

Sample collection happened between December 2012 and April 2014 via outreach and online sampling among MSM. Test results were communicated through a secured website. HIV tests were executed in the laboratory. Each reactive sample needed to be confirmed using state-of-the-art confirmation procedures on a blood sample. Close follow-up of participants who did not pick up their results, and those with reactive results, was included in the protocol. Participants were asked to provide feedback on the methodology using a short survey.

**Results:**

During 17 months, 1071 tests were conducted on samples collected from 898 men. Over half of the samples (553/1071, 51.63%) were collected during 23 outreach sessions. During an 8-month period, 430 samples out of 1071 (40.15%) were collected from online sampling. Additionally, 88 samples out of 1071 (8.22%) were collected by two partner organizations during face-to-face consultations with MSM and male sex workers. Results of 983 out of 1071 tests (91.78%) had been collected from the website. The pickup rate was higher among participants who ordered their kit online (421/430, 97.9%) compared to those participating during outreach activities (559/641, 87.2%; *P*<.001). MSM participating during outreach activities versus online participants were more likely to have never been tested before (17.3% vs 10.0%; *P*=.001) and reported more sexual partners in the 6 months prior to participation in the project (mean 7.18 vs 3.23; *P*<.001). A total of 20 participants out of 898 (2.2%) were confirmed HIV positive and were linked to care. Out of 1071 tests, 28 (2.61%) with a weak reactive result could not be confirmed, and were thereby classified as false reactive results. Most of the 388 participants who completed posttest surveys (388/983, 39.5%) were very positive about their experience. The vast majority (371/388, 95.6%) were very satisfied, while 17 out of 388 (4.4%) reported mixed feelings.

**Conclusions:**

Despite a high yield and a considerable number of false reactive results, satisfaction was high among participants. The project helped us to reach the target population, both in numbers of tests executed and in newly diagnosed HIV infections. Further optimization should be considered in the accuracy of the test, the functionalities of the website (including an online counseling tool), and in studying the cost effectiveness of the methodology.

## Introduction

HIV remains an important public health problem. In the European Union, 29,157 new HIV infections were reported in 2013, an incidence of 5.7 per 100,000 inhabitants [1]. A total of 42% of new infections were among men who have sex with men (MSM). Countries with the highest incidence were Estonia (24.6 per 100,000 inhabitants), Latvia (16.8), Portugal (10.4), and Belgium (10.0) [1].

Promoting HIV testing is an integral part of the 90-90-90 Joint United Nations Programme on HIV/AIDS (UNAIDS) plan to end the AIDS epidemic by 2030. In terms of this plan, 90% of all people living with HIV should know their HIV status, 90% should be on treatment, and 90% of these should be virologically suppressed [2]. Part of the rationale for this strategy is that intensified HIV testing contributes to earlier commencement of antiretroviral therapy (ART) which in turn leads to reduced HIV transmission via reducing the HIV viral load [3]. HIV diagnosis also leads to behavioral changes in sexual risk taking in a majority of newly diagnosed persons [4].

Increased HIV testing among those at risk is a key way of achieving the required target of 90% of HIV-infected people knowing their HIV status. The traditional HIV test is offered voluntarily and confidentially by a medically trained health care professional in a health care setting with a strong emphasis on the patient’s informed consent [5]. Counseling and test results are provided by trained health care workers during a face-to-face consultation [6]. This approach may remain the standard for most people. However, HIV has reached endemic proportions among MSM in the industrial world, with incidence rates of 2 to 3% per year, and prevalence between 10 and 30% [7]. These men are generally well informed about HIV [8] and in certain groups of MSM, pretest counseling was found to be "repetitive" and "unnecessary" [9]. For these men, alternative HIV testing strategies can be considered. One study found that oral fluid testing is preferred by MSM above giving blood samples [10]. It has also recently been found to be reliable for diagnostic use in groups with an HIV prevalence over 1% [11] like MSM, and several research projects in clinical settings have shown promising results for HIV tests on oral fluid samples [12,13].

Rapid HIV tests, decentralized HIV testing (ie, outreach and community-based testing), and self-testing are additional alternatives. Rapid tests are used in a variety of settings, including primary health care settings [14,15], emergency departments [16,17], and in dental clinics [18]. Their advantage is that clients receive their results at the time of their visit [19]. A major disadvantage is that in low-HIV-prevalence settings they give a relatively high proportion of false positive results [13]. Outreach testing targeting MSM has been implemented in clubs, bars, and bath houses [20-22], as well as at large-scale events, such as Gay Pride festivals [23]. Community-based testing among MSM is increasing in recent years [24,25]. In Europe, an increasing number of community-based testing centers (ie, Checkpoints) have been established [26,27]. Self-tests for HIV that can be ordered through the Internet are the most recent development in this field [28]. The only US Food and Drug Administration (FDA)-licensed oral fluid-based rapid test is OraQuick ADVANCE Rapid HIV-1/2 Antibody test [29]. The FDA approved the use of this test for home use in July 2012 [30], despite its relatively high risk of false positive results [31], especially among lower-risk groups [13]. The quality of other test kits that can be ordered online is largely unknown due to their lack of certification. The advantages of self-testing include increased convenience and heightened privacy [28]. The difficulty of ensuring linkage to care in the case of a positive result is a weakness of these tests. In a recent review, supervised and unsupervised self-testing strategies were found to be highly acceptable and preferred, but all studies lacked an evaluation of posttest linkage to counseling and care [32]. Internet-based testing can therefore be an alternative, where posttest linkage is part of the process. The willingness to use Internet-based HIV-testing strategies was high in recently published quantitative and qualitative studies [33,34]. To address MSM’s testing needs, we developed the Swab2know project [35]. This project combines two strategies to increase HIV-testing uptake among MSM: outreach HIV-test sessions and free online testing. In both strategies, samples are collected using oral fluid collection devices and test results are communicated via secured website.

This nonrandomized, prospective descriptive study aimed at detecting new HIV cases among groups at risk for HIV/sexually transmitted infection (STI) acquisition. The secondary objective was to assess the acceptability and feasibility of an HIV-testing strategy with the use of self-administered oral fluid samples collected through outreach and online activities and Web-based delivery of test results.

## Methods

### Population and Settings

The project targeted two main risk groups for HIV infection in Belgium: MSM and sub-Saharan African migrants (SAM) socializing in community venues. Only the data from MSM were used for this analysis; the findings for SAM will be published elsewhere.

Outreach sessions for MSM were organized in five types of venues, mostly situated in Antwerp: saunas/bathhouses, fetish scene venues, dancing/discotheque venues, outreach sessions organized during the World Outgames in Antwerp targeting athletes and supporters, and large-scale gay events.

Inclusion criteria were that participants had to identify themselves as MSM and be 18 years of age or older. Additional criteria were that participants had to be accepting of oral fluid sampling, sign informed consent forms, provide minimal information, and understand that the test, if positive, would only be strongly indicative of HIV infection. Participants who were not willing to provide a mobile phone number or email address, were under 18 years of age, or were unable to sign the informed consent form were excluded from this project and redirected to standard testing facilities.

### Website

A website, swab2know.be, has been specifically designed for this project [35]. The main aim of the website is to provide a platform where visitors can find information, prevention messages, order test kits, and collect their test results. This website is secured by means of the Secure Sockets Layer protocol, and holds a security certificate provided by Belnet—Belnet is the federal government organization that provides high-bandwidth Internet connection and services to Belgian universities, research centers, and government departments. The certificate confirms the identity of, and encrypts the communication between, the Swab2know Web server and the computer where the information is requested.

All online materials described in the Methods section are available through the study website [35].

### Sampling Procedures

Outreach sessions took place in various MSM leisure venues. Field workers of Sensoa, a local prevention organization, announced the Swab2know session at the entrance. If clients decided to participate, they visited the Swab2know team in a separate room. All materials were available in Dutch, English, and French. After being informed and signing the informed consent (IC) form, baseline data using a self-administered pen-and-paper questionnaire (see Multimedia Appendix 1) were gathered from participants and an online account was created. Each account was unique and linked with an email address and phone number. A generated password was sent automatically to the participant’s email address. The oral fluid samples were self-collected by the participants under the supervision of study staff. All samples were identified by a unique sample code, which linked the sample with the personal account, the IC, and baseline data. Samples were kept at room temperature and were brought to the laboratory on the next working day.

Online recruitment happened on the website by occasional visitors who created an account and provided their email address and phone number. The project was advertised by prevention organizations and through articles and announcements in dedicated media, including gay-oriented websites and magazines and a Swab2know Facebook account. Participants provided consent by accepting the terms of the study. A sampling kit identified with a unique sample code was sent to the Belgian address of their choice. Participants took the oral fluid sample after having seen a short educational video on the website. Samples were sent to the lab with a prepaid envelope. The participants could also opt to collect their results during a face-to-face consultation.

### HIV Test

The validation of the accuracy of the test has previously been evaluated in our AIDS Reference Laboratory at the Institute of Tropical Medicine (ITM) [10]. Each sample underwent a two-step HIV-test procedure. First, all samples were tested for HIV using Genscreen HIV-1/2 Version 2 by Bio-Rad (Marnes-la-Coquette, France) [36]. The results were classified as strong, weak, or nonreactive. In a second step, all nonreactive samples were checked for sample quality using a human IgG detection test. The quality of the oral fluid samples was measured using the IgG enzyme-linked immunosorbent assay (ELISA) quantification kit (Human IgG ELISA Immunology Consultants Laboratory, Inc, Portland, OR, USA). If the sample contained more than 3500 ng total IgG/mL, the nonreactive result was considered valid and ready to be communicated. Prior to uploading them onto the website, each result of the HIV test performed was technically validated by two persons, individually.

### Communication of the HIV Test Results

Once the results of the HIV tests were known in the laboratory, they were uploaded onto the website. Upon uploading, participants received an email indicating that their result was available. Participants received one of four standardized messages (for full messages, see Multimedia Appendices 2-5): (1) a strong reactive test result, strongly indicating HIV infection, to be confirmed by a blood sample, (2) a weak reactive result, indicating a probable false positive result or an early infection, to be confirmed by a blood sample, (3) a nonreactive result, indicating the absence of HIV infection, taking into account a window period of 3 months, and (4) an invalid result, with the suggestion to repeat the oral fluid sample or to take a state-of-the-art HIV test. In the case of a reactive result, a mobile phone number was provided for emergency counseling by a trained paramedic.

Participants who did not check their results were contacted by phone or email. All participants with a reactive result were contacted by phone within 24 hours of having read their results. The purpose of the call was to offer counseling and to arrange a further confirmation test and guarantee linkage to care. If confirmation did not take place at the organizing health care center, participants were contacted after the confirmation procedure to collect the final result.

### Repeated Testing

Participants with a nonreactive test result, both through outreach and online participation, were offered the possibility to order a sampling package to be delivered to a Belgian address every 4 to 6 months, allowing frequent and repeated testing. For this purpose, a reminder email was sent 4 to 6 months after participation to the email address linked with the personal account.

### Acceptability of the Methodology

In the delivery message of the test result, participants were asked to fill out an online self-administered survey. In this survey, participants provided information on their impression of the project as a whole (not good, mixed feelings, good) and whether they would participate again in the future (no, not sure, without hesitation).

### Statistical Analysis

Statistical analysis was performed using IBM SPSS version 22. Descriptive and univariate analyses were carried out. Chi-square tests were used for categorical variables and independent samples *t* tests for continuous variables. A significance level of 5% was applied.

### Ethical Considerations

The methodology was conceptualized in close collaboration with community-based prevention organizations targeting MSM and African communities.

Ethical approval was obtained from the Institutional Review Board at the ITM and the University Hospital in Antwerp.

## Results

### Number of Performed Tests

In a period of 17 months (December 2012-April 2014), 1082 tests were executed on samples collected through outreach activities and online ordering of sampling kits. A total of 11 tests were excluded from the analysis because the participants disclosed their HIV-positive status during the baseline survey. Data from 1071 tests from 898 participants were used for this analysis. A total of 4 persons participated 4 times, 16 participated 3 times, and 129 participated twice in this period. A total of 53 persons participated during outreach activities and ordered a sampling kit later in the project.

During 23 outreach sessions, 553 out of a total 1071 (51.63%) samples were collected. These sessions were organized in saunas/bathhouses (5 sessions), fetish scene venues (4 sessions), dancing/discotheque venues (8 sessions), during the World Outgames in Antwerp targeting athletes and supporters (3 sessions), and at other gay events (3 sessions). Additionally, 88 samples out of 1071 (8.22%) were collected by two partner organizations who used the project’s methodology to facilitate HIV testing within their regular activities during face-to-face consultations with MSM and male sex workers. For the analysis, these samples were added to the *outreach* group. These 641 samples out of 1071 (59.85%) were collected from 609 men. From September 1, 2013, we offered people the possibility of ordering a sampling kit through the website. In the subsequent 8-month period, 430 samples out of 1071 (40.15%) were ordered online by 289 participants.

### Participant Characteristics

A description of the population with a comparison between the outreach and the online population is presented in Table 1.

**Table 1:** Characteristics of study participants.

Characteristic		Online participants (n=430), mean (SD) or n (%)	Outreach participants (n=641), mean (SD) or n (%)	*P*
Age in years, mean (SD)		34.3 (10.2)	33.5 (11.4)	.25
**Sexual** **partners, n (%)**				.07
	Men	397 (92.3)	524 (81.7)	
	Men and women	30 (7.0)	52 (8.1)	
	Women^a^	3 (0.7)	0 (0)	
Has a general practitioner/family doctor, n (%)		389 (90.5)	558 (87.1)	.10
Never tested for HIV, n (%)		43 (10.0)	111 (17.3)	.001
Number of sexual partners in past 3 months, mean (SD)		3.23 (5.02)	7.18 (13.53)	<.001

^a^All participants reporting sexual contacts with women answered "transgender" on the question for gender.

### Results Communication

The vast majority (1057/1071, 98.69%) of test results were delivered through the website. A total of 14 out of 1071 results (1.31%) were delivered either by phone (mainly because the participants did not have access to email or Internet) (8/1071, 0.75%) or during a face-to-face consultation (6/1071, 0.56%). All results that were not communicated through the website stemmed from samples collected through outreach activities.

Overall, the results of 983 out of 1071 (91.78%) tests were effectively collected from the website. The pickup rate was significantly higher when the test had been ordered online (421/430, 97.9%) compared to the test performed during outreach activities (559/641, 87.2%; *P*<.001).

**Figure 1:**
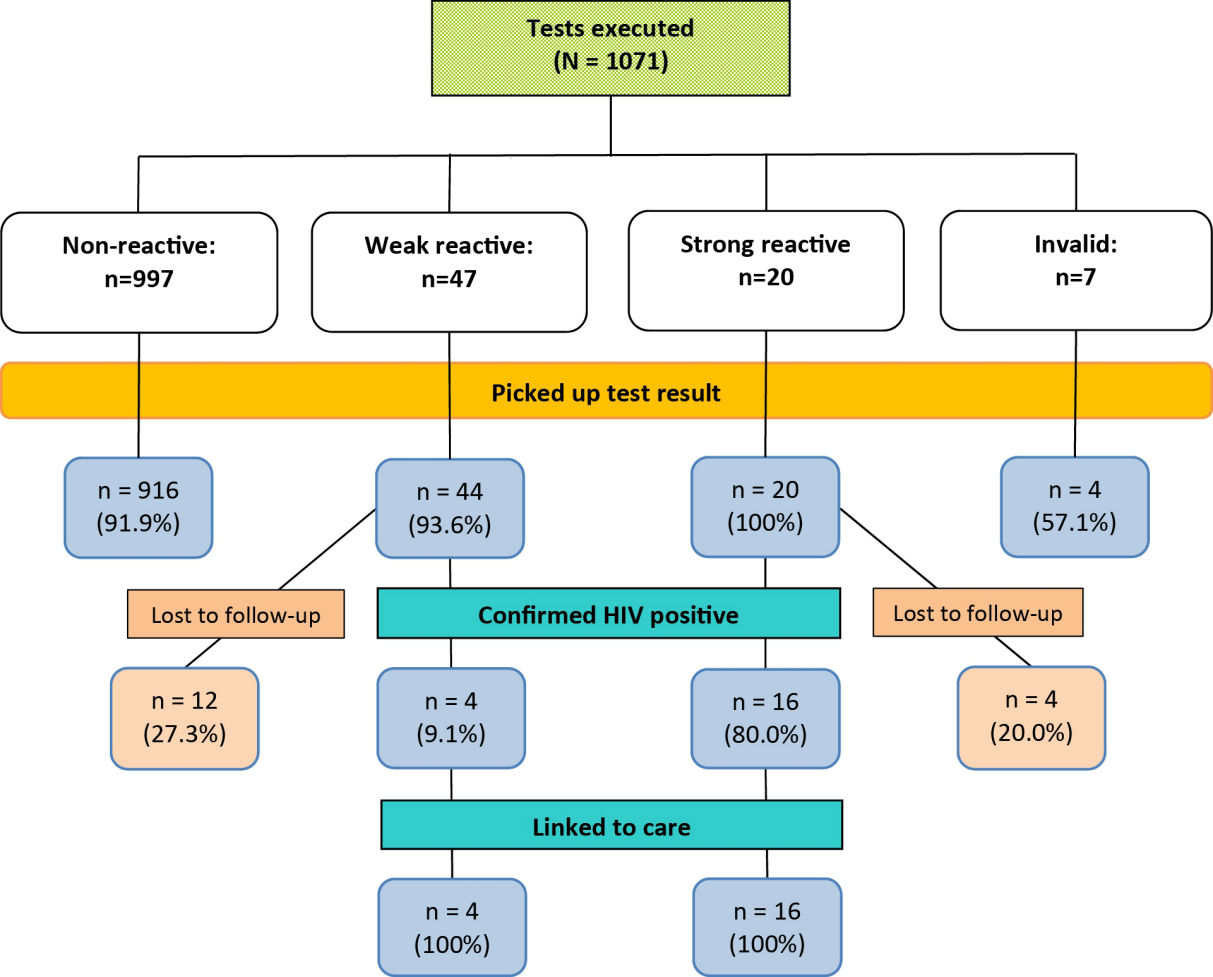
Flowchart of test results, confirmation, and follow-up for 1071 tests executed among MSM in the Swab2know study.

Figure 1 shows the number of nonreactive, weak reactive, and strong reactive test results, with confirmation test results from blood and linkage to care.

A total of 28 out of 44 (64%) weak reactive test results were not confirmed HIV positive, and were thereby classified as *false reactive* results.

Overall, 20 participants were confirmed as newly diagnosed with HIV and all of them were linked to care; this represents 2.2% (20/898) of all participants tested. A total of 6 newly diagnosed participants ordered their sampling kit online which put the new HIV infection rate in this group at 2.1% (6/289) while 14 were detected during outreach sessions; the new HIV infection rate for this group was 2.3% (14/609). This difference was not statistically significant (*P*=.83).

### Acceptability

Of the 983 participants who collected their test results, 388 (39.5%) completed posttest surveys. The vast majority of participants (371/388, 95.6%) reported being very satisfied with the process while 17 out of 388 (4.4%) experienced mixed feelings taking part in the project. Whereas 48 out of 388 participants (12.4%) reported they would consider taking part again, most of the respondents (337/388, 86.9%) reported they would do so "without hesitation." Of the 388 respondents, 3 (0.8%) reported that they would not participate again in the future. One of them had been diagnosed with HIV through the project, which makes future participation redundant.

## Discussion

### Principal Findings

Results from recent surveys show that HIV self-testing is gaining momentum within the MSM community across the world [34,37]. This manuscript describes a low-threshold HIV-testing strategy combining oral fluid self-sampling, HIV testing in a specialized AIDS reference laboratory, and result delivery through a secured website with a solid linkage-to-care strategy.

Compared to the bulk of research on HIV self-testing and home-based testing, little has been published on the combination of self-sampling and the remote delivery of the test result. A recent project in the United Kingdom used a similar methodology to our project, but they performed tests on dried blood samples. Negative results were disclosed by text message while positive results were communicated by phone. They found a comparable rate of newly diagnosed HIV infections, successful linkage to care, and participant satisfaction [38].

We specifically chose oral fluid collection devices given their potential advantages: convenient and painless to collect, ideal for self-sampling, and very little risk of contamination during collection and sample transport. The potential problems associated with oral fluid testing are a lower sensitivity especially in detecting early infections and the fact that one cannot perform a confirmation test on the same sample because this needs to be done on a blood sample.

We also decided not to use a rapid test during outreach sessions despite good results described in similar settings [21,22]. This choice was motivated by two reasons. First, when a session is organized in leisure venues, the idea of receiving an HIV diagnosis on the spot could prevent clients from participating. Second, a reactive result requires thorough counseling and support, which are hard to deliver in these types of venues, especially when other participants are waiting to be tested. Our alternative strategy was to deliver the results via an online tool.

Acceptability and intended uptake of Internet-based HIV/STI-screening programs are high among high-risk groups in various settings [34,39]. Nevertheless using a website to communicate HIV test results has, to the best of our knowledge, not been reported before. It has several advantages over other communication strategies used in similar projects. The participant, not the health care provider, decides when to pick up the result. It is less time consuming and less intrusive than a phone call. It offers the possibility to provide information in addition to the HIV test result, such as information on the test window period, the importance of testing for other STIs, and the need to confirm the result in case of a reactive test. Compared to text messages, website communication opens up possibilities to develop automated counseling strategies tailored to the test results and the patient's profile in the future.

The self-sampling procedure produced samples of acceptable quality. A small minority of participants (all through online testing) provided an invalid sample. Satisfaction with the project was very high among participants; however, given the incomplete response rate to the satisfaction survey, we cannot exclude the possibility of a selection bias.

The project helped us to reach the target population, both in terms of the number of tests executed, and in the number of newly diagnosed HIV infections. The percentage of newly diagnosed participants (2.2%) was higher than expected. As recently reported, 6% of Belgian MSM tested in a variety of nightlife settings are HIV positive [40], of whom 14.3% are unaware of their HIV-positive status. Applying these figures, we expected to diagnose 9 new HIV infections (14.3% of 6% of 1071 tests) compared to the 20 new HIV diagnoses in this project. This may be an indication that we succeeded in attracting the population at highest risk of acquiring HIV. All new cases were successfully linked to HIV care, which is a crucial aspect of the HIV treatment cascade, and a great asset of the project compared to self-testing. Moreover, with a yield of 2.2% of participants (20/898) newly diagnosed with HIV in this project, its method can be considered as cost effective—HIV testing in populations where the prevalence is greater than 0.1% is considered cost effective [41,42].

An additional benefit of the project was that the partners from 3 participants were newly diagnosed in the organizing health center during the course of the project. They were not included in this analysis.

The proportion of participants who were never tested before was considerable. Of 898 participants, 154 (17.1%) answered that they had never been tested for HIV before. This percentage indicates that online and outreach testing may facilitate HIV testing for MSM who experience difficulties in taking a test using the existing structures, and therefore do not get tested.

### Limitations

As observed previously with oral fluid testing protocols, the proportion of *false reactive* test results was substantial (2.7%) [13]. Taking into account the impact of a reactive result on people’s lives, these *false reactive* results should be minimized. Despite this problem, 4 of 6 participants with a *false reactive* result who provided feedback using the acceptability questionnaire answered that they were "very satisfied" with the project and would "without hesitation" participate again. The other 2 participants reported "mixed feelings" about the project: one said he would not and one said he would consider participating again. It remains crucial that participants with a weak reactive result see a physician to confirm or refute the result. A minority of participants with a weak reactive result were confirmed HIV positive (4/32, 13%).

A considerable number of participants were lost to follow-up in the course of the project. Whereas a loss to follow-up does not mean that participants were not linked to care (some may have visited their general practitioner), we should aim to minimize this proportion.

The yield of this screening project was high; however, contacting and motivating participants to pick up their results required more intensive follow-up than expected. Although the workload for the paramedical staff was much less than with the standard-of-care counseling method, this aspect should not be underestimated in such projects. Further studies should investigate whether such strategies are cost effective in detecting new HIV infections in high-risk groups.

### Next Steps

We plan to continue the project in the coming years, with an increased emphasis on Internet-based testing and repeated testing for participants, as well as strong collaboration with community-based and prevention organizations to guide MSM toward the Swab2know project. On the basis of this experience, our methodology will be refined. First, the online counseling tool will be further developed and refined to support participants, with an increased emphasis on those with a reactive result. Comparable e-counseling tools have been developed and implemented in primary care [43]. This could complement, and to some extent replace, the phone counseling, thereby reducing the staff workload. Second, we hope to reduce the number of false reactive results by the use of newly developed point-of-care oral fluid tests. Third, expanding the scope of sexually transmitted infections tests may improve the attraction of the project among MSM. One could consider performing syphilis or hepatitis C serology on oral fluid samples, or nucleic acid tests for the detection of chlamydia and gonorrhea on self-collected urethral and anal samples [44], allowing a comprehensive STI checkup.

From a societal point of view, a legal framework needs to be developed. Self-testing is not officially recognized in Belgium: neither are online testing nor self-sampling activities.

In conclusion, we demonstrated that a low-threshold HIV-testing strategy combining self-sampling with oral fluid and online result delivery was acceptable. The HIV infection rate was higher than expected and the linkage to care was good. This strategy empowers individuals to manage their health, but at this stage it should be reserved for high-risk groups such as MSM where the incidence of HIV is high.

## Appendix

Multimedia Appendix 1 Baseline questionnaire filled in by all participants.

Multimedia Appendix 2 Full message that was communicated in the case of a strong reactive test result.

Multimedia Appendix 3 Full message that was communicated in the case of a weak reactive test result.

Multimedia Appendix 4 Full message that was communicated in the case of a nonreactive test result.

Multimedia Appendix 5 Full message that was communicated in the case of an invalid test result.

## Abbreviations

ART: antiretroviral therapy
ELISA: enzyme-linked immunosorbent assay
FDA: US Food and Drug Administration
IC: informed consent
ITM: Institute of Tropical Medicine
MSM: men who have sex with men
SAM: sub-Saharan African migrants
STI: sexually transmitted infection
UNAIDS: Joint United Nations Programme on HIV/AIDS
