# Supplementary figures and images for: Swab2know: An HIV-Testing Strategy Using Oral Fluid Samples and Online Communication of Test Results for Men Who Have Sex With Men in Belgium
Source: J Med Internet Res. 2015 Sep 1;17(9):e213. doi: 10.2196/jmir.4384 (PMC4642797; doi:10.2196/jmir.4384)

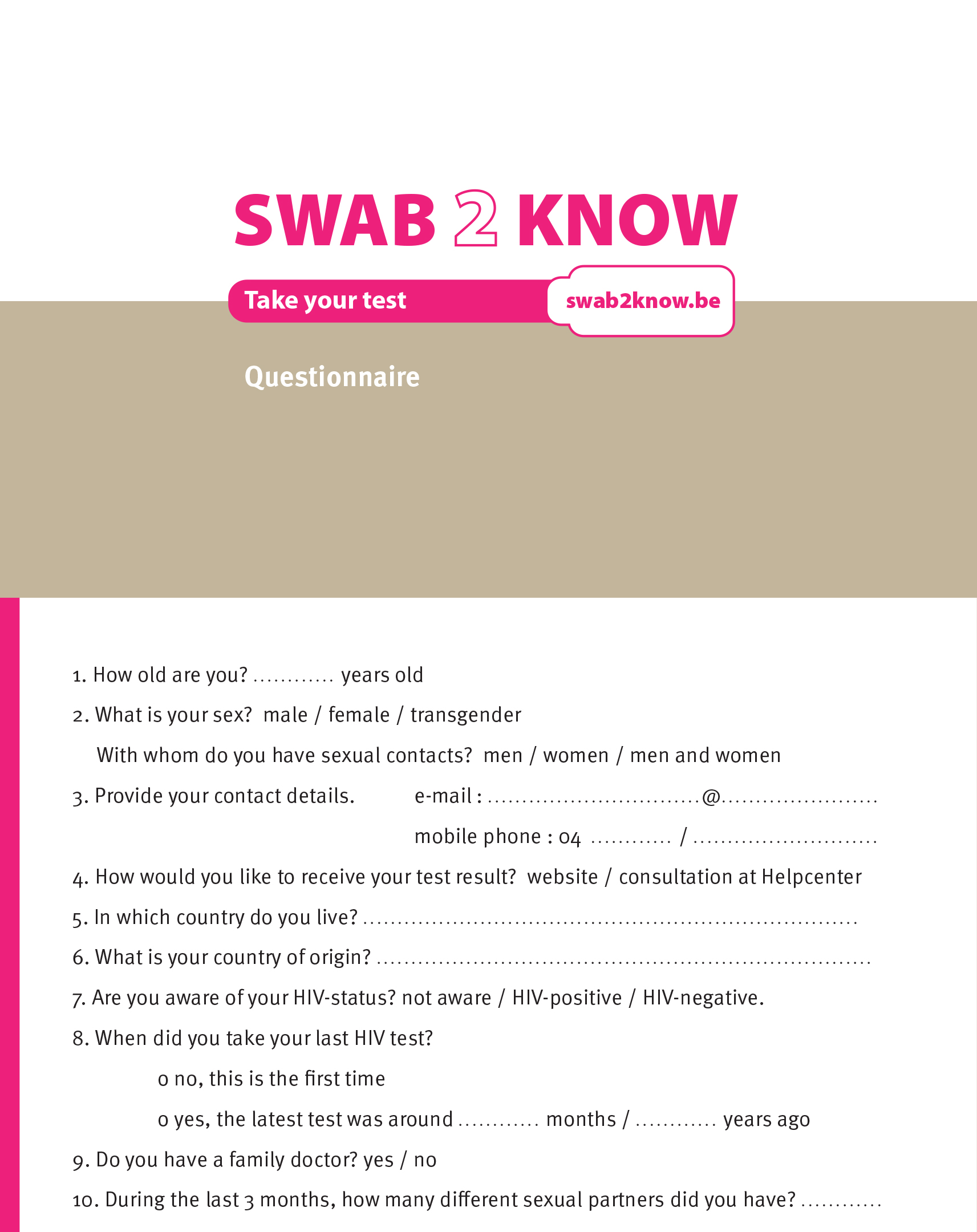

Supplement: Multimedia Appendix 1 [file jmir_v17i9e213_app1.jpg]

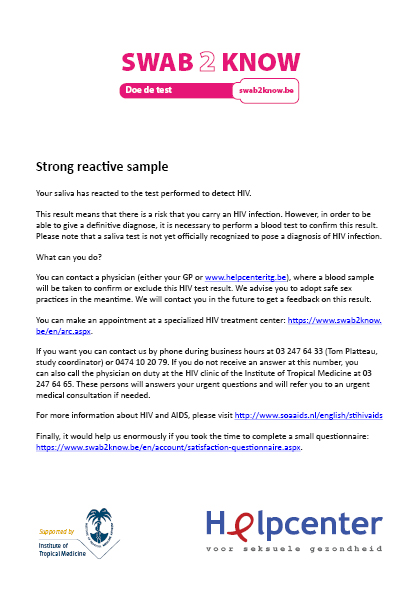

Supplement: Multimedia Appendix 2 [file jmir_v17i9e213_app2.jpg]

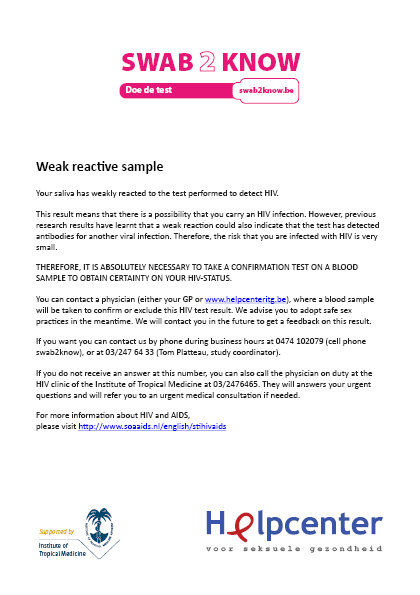

Supplement: Multimedia Appendix 3 [file jmir_v17i9e213_app3.jpg]

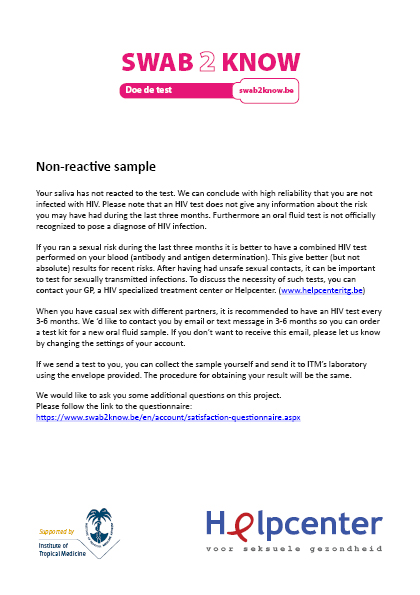

Supplement: Multimedia Appendix 4 [file jmir_v17i9e213_app4.jpg]

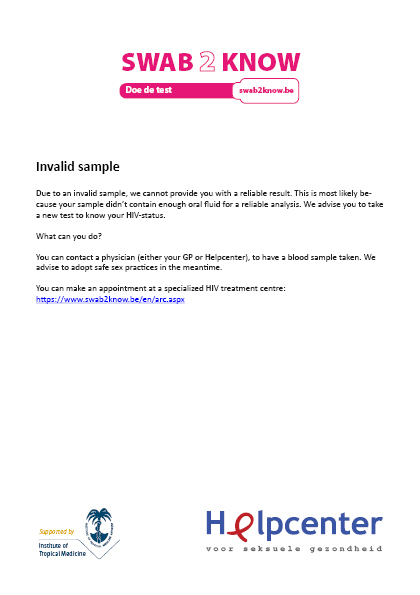

Supplement: Multimedia Appendix 5 [file jmir_v17i9e213_app5.jpg]
